# Supplementary material for: Splice-Junction-Based Mapping of Alternative Isoforms in the Human Proteome
Source: Cell Rep. Author manuscript; Available in PMC 2020 Jan 15. (PMC6961840; doi:10.1016/j.celrep.2019.11.026)
Supplement: 3 [file NIHMS1546469-supplement-3.zip › DF2/PXD009737/TestisGluC-47-P02751-YTIYVIALKNNQKSEPLIGRKKTGQE.pdf]

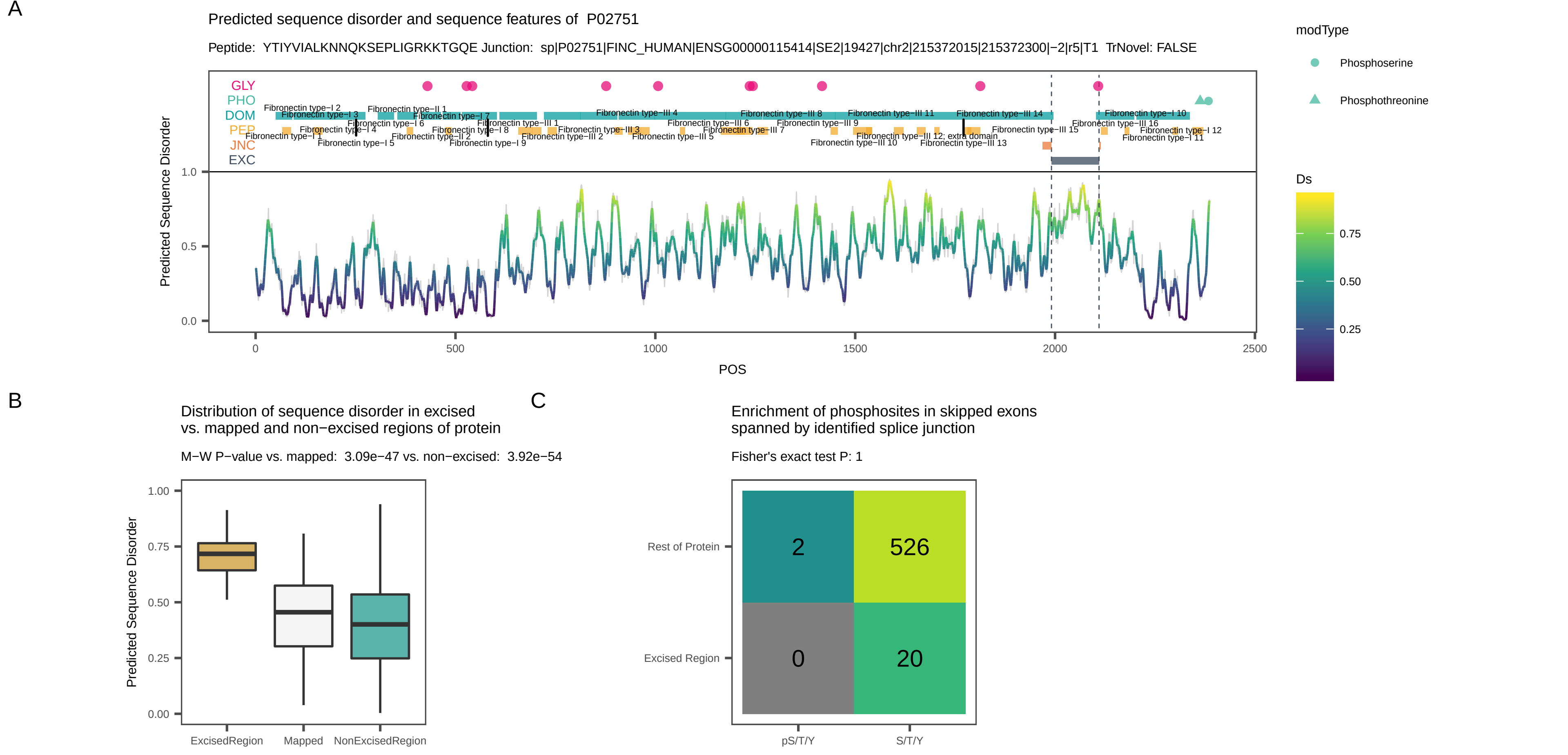
A
Predicted sequence disorder and sequence features of P02751
Peptide: YTIYVIALKNNQKSEPLIGRKKTGQE Junction: sp|P02751|FINC_HUMAN|ENSG00000115414|SE2|19427|chr2|215372015|215372300|−2|r5|T1 TrNovel: FALSE
GLY
PHO
DOM
PEP
JNC
EXC
Predicted Sequence Disorder
POS
modType
Phosphoserine
Phosphothreonine
Ds
B
Distribution of sequence disorder in excised vs. mapped and non−excised regions of protein
M−W P−value vs. mapped: 3.09e−47 vs. non−excised: 3.92e−54
Predicted Sequence Disorder
ExcisedRegion
Mapped
NonExcisedRegion
C
Enrichment of phosphosites in skipped exons spanned by identified splice junction
Fisher's exact test P: 1
Rest of Protein
Excised Region
2
526
0
20
pS/T/Y
S/T/Y
